# Efficient Detection of Oligodendroglioma With 1p/19q Codeletion Mutation via Methionine PET Imaging: A Promising Diagnostic Approach

**DOI:** 10.7759/cureus.77826

**Published:** 2025-01-22

**Authors:** Edgardo de Jesús Mateo-Nouel, Michel G Mondragón-Soto, Carlos F Nicolás-Cruz, Eliezer Villanueva-Castro, Luis A Rodriguez Hernandez, Ignacio Reyes-Moreno, Jesús A Violante Villanueva, Roberto A de Leo-Vargas, Sergio M Jiménez, Alberto Gonzalez Aguilar

**Affiliations:** 1 Neurosurgery, Instituto Nacional de Neurología y Neurocirugía Manuel V. Suárez, Mexico City, MEX; 2 Neuro-Oncology, Instituto Nacional de Neurología y Neurocirugía Manuel V. Suárez, Mexico City, MEX; 3 Neurology, Instituto Nacional de Neurología y Neurocirugía Manuel V. Suárez, Mexico City, MEX

**Keywords:** 1p19q codeletion, glioma, oligodendroglioma, pet met, pet methionine

## Abstract

Background

Oligodendrogliomas are a distinct subtype of gliomas frequently characterized by the 1p/19q codeletion and isocitrate dehydrogenase (*IDH*) gene mutations, both associated with improved therapeutic response and prolonged survival. These genetic alterations modulate the transsulfuration pathway, leading to increased methionine uptake by tumor cells. Positron emission tomography (PET) with 11C-labeled methionine (MET PET) leverages these metabolic changes, providing a noninvasive means to distinguish oligodendrogliomas and predict the 1p/19q codeletion presence. This study evaluates the diagnostic potential of MET PET in detecting 1p/19q deletions and quantifying SUV max (maximum standardized uptake value) to evaluate metabolic activity in newly diagnosed oligodendrogliomas, emphasizing the value of advanced imaging in guiding targeted clinical management.

Methods

We performed a retrospective chart review of pediatric and adult patients treated between 1999 and 2010, a period selected to capture evolving clinical protocols and advancements in imaging techniques. This timeframe maximized data availability and provided a longitudinal perspective on how shifts in diagnostic and therapeutic strategies may have influenced outcomes. All participants underwent MET PET scans and subsequent oligodendroglioma resections, with follow-up data extending until 2010. Relevant information, including demographics, clinical details, and glioma-specific mutations, was extracted from clinical records. Cases without histological confirmation or missing genetic results (1p/19q codeletion, *IDH*) were excluded to safeguard data integrity and limit bias. Both univariate and multivariate linear regression analyses were employed to assess the relationship between MET PET findings (SUV max) and these genetic alterations, aiming to clarify the predictive value of PET imaging in tumor genetics.

Results

Among the 85 oligodendroglioma patients analyzed (median age 50 ± 3 years), 47.1% (n = 40) harbored the 1p/19q codeletion, whereas 52.9% (n = 45) did not. The median SUV max was significantly higher in patients lacking the codeletion (3.7, IQR: 2.9-4.4) than in those with it (2.2, IQR: 1.8-2.6; p < 0.001). A Mann-Whitney U test confirmed the discrepancy (U = 189, z = -6.261, p < 0.0001). Further analysis using a multiple linear regression model indicated that the absence of the 1p/19q codeletion and an elevated Ki-67 index collectively predicted higher SUV max (F(1, 82) = 10.43, p < 0.0001), accounting for approximately 42.2% of the variability in SUV max.

Conclusions

The findings from this study underscore the utility of the MET PET scan not only as a diagnostic tool for identifying the presence of the 1p/19q deletion in patients with oligodendrogliomas but also for evaluating tumor metabolism through SUV max measurements. The scan's ability to distinguish tumor from necrosis based on metabolic activity enhances its clinical value, providing critical insights for optimal patient management. The data suggest that patients with higher SUV max are more likely to lack the 1p/19q deletion, a finding that could significantly influence treatment decisions and prognostic assessments. Given these results, MET PET scans represent a potent tool in refining diagnostic and follow-up strategies for oligodendroglioma, guiding more targeted and effective therapeutic approaches.

## Introduction

Gliomas are the most common primary malignant brain tumors, encompassing a wide spectrum of neoplasms with varying degrees of aggressiveness and prognosis [1]. Among these, oligodendrogliomas are notable for their distinct clinical, pathological, and genetic features, particularly the 1p/19q codeletion [2]. This genetic alteration is strongly associated with improved responses to chemotherapy and radiotherapy, leading to extended survival rates. Additional molecular markers often accompany the codeletion, further influencing clinical outcomes [3]. Unlike astrocytomas and ependymomas, oligodendrogliomas typically exhibit heightened sensitivity to chemotherapy, resulting in a more favorable prognosis [4].

The genetic landscape of oligodendrogliomas is defined by the codeletion of the short arm of chromosome 1 (1p) and the long arm of chromosome 19 (19q), known as the 1p/19q codeletion [2]. This alteration is a hallmark of oligodendrogliomas and is strongly associated with enhanced treatment responses and prolonged survival. Moreover, the 1p/19q codeletion often co-occurs with other molecular markers that influence clinical outcomes. These include MGMT (unmethylated methylguanine methyltransferase) promoter methylation, predictive of sensitivity to alkylating agents; *IDH1* (isocitrate dehydrogenase) mutations, common in most low-grade gliomas and secondary high-grade gliomas; and the CpG island methylator phenotype (G-CIMP), which correlates with a more favorable prognosis [3].

Beyond their genetic characteristics, oligodendrogliomas exhibit distinct metabolic alterations that further differentiate them from other gliomas. Notably, there is an altered metabolism of cystathionine, a precursor to cysteine and glutathione, which are critical for cellular antioxidant defenses [5]. In *IDH*-mutated and 1p/19q codeleted gliomas, increased expression of cystathionine-β-synthase (CBS), the enzyme catalyzing the first step of the transsulfuration pathway, has been associated with improved patient outcomes [6]. These findings suggest that specific metabolic pathways modulated by genetic alterations could serve as potential targets for metabolic-based therapies.

Advanced neuroimaging, particularly positron emission tomography (PET) with 11C-labeled methionine (MET), has become invaluable for diagnosing oligodendrogliomas. By delineating the metabolic extent and functional status of lesions, MET PET enhances tumor-type differentiation and facilitates precise evaluation of treatment responses, addressing key diagnostic challenges [7,8]. Moreover, this modality is instrumental in detecting tumor recurrence, distinguishing neoplastic tissue from necrosis, gauging tumor aggressiveness, and monitoring metabolic activity. Consequently, MET PET is an essential tool in guiding surgical planning and optimizing therapeutic strategies in oligodendroglioma management.

This study aims to further elucidate the utility of MET PET in oligodendrogliomas by examining the relationship between MET uptake and critical diagnostic and prognostic markers, including histological features, the Ki-67 proliferation index, and the presence of the 1p/19q codeletion [2,3].

## Materials and methods

This study assessed the diagnostic utility of MET PET imaging in a cohort comprising both pediatric and adult patients presenting with previously untreated cerebral lesions suggestive of gliomas. All participants underwent comprehensive preoperative evaluation using MET PET to confirm and characterize these lesions further, enhancing our understanding of their metabolic activity and potential malignancy before surgical intervention.

In the development of this retrospective study, information extracted from medical records was used. It is important to note that all medical records were handled confidentially and were not deanonymized. The protection of the identity of the patients was guaranteed, complying with the relevant ethical and legal regulations for health research.

Inclusion criteria

Preoperative MET PET Assessment

During the initial diagnostic workup, each patient underwent a MET PET scan to evaluate cerebral lesions indicative of oligodendroglioma. This imaging provided critical insights into lesion metabolism and guided surgical planning.

Surgical Intervention

All included patients were scheduled for surgical resection of the identified lesions. This approach enabled direct correlations between MET PET findings and histopathological results.

Molecular Marker Analysis

Eligibility required the availability of molecular data for *IDH* mutations and 1p/19q codeletion status, both of which are essential for glioma classification and prognosis.

Postoperative Pathological Confirmation

The lesions underwent detailed histopathological examination post-resection to validate preoperative MET PET findings and integrate imaging data with clinical and molecular characteristics.

Patients

This study analyzed a cohort of 85 patients diagnosed with oligoastrocytoma or oligodendroglioma, with diagnoses confirmed through comprehensive histopathological evaluation. Advanced imaging modalities, including MRI and CT, were employed to accurately localize and characterize brain lesions. Additionally, each patient underwent MET PET imaging using a Siemens 11 MeVp CT (Munich, Germany) scanner. These scans were conducted at the Instituto Nacional de Neurología y Neurocirugía Manuel Velasco Suárez in Mexico City, Mexico, over a study period spanning from 1999 to 2010, providing detailed metabolic insights to complement structural imaging findings.

The clinical application of the MET PET/CT tracer in this research was approved by the Institutional Ethical Committee, ensuring that all experimental protocols adhered to the highest ethical standards. Written informed consent was obtained from all participants, affirming their voluntary participation and comprehension of the study's objectives and methodologies.

MET PET/CT scans were integral to the study, offering critical insights into the metabolic activity of the brain lesions. The standardized uptake value (SUV) of MET, a metric indicating the relative uptake of the tracer by the brain tissue, was evaluated semiquantitatively. This measure is vital for distinguishing different types of brain lesions based on their metabolic profiles, thus providing essential diagnostic and prognostic data.

Data for the study were meticulously gathered from medical records and managed through the local Picture Archiving and Communication System (PACS). This system facilitated the efficient handling, retrieval, and analysis of imaging data alongside patient records, streamlining the research process and ensuring data integrity.

Inter-laboratory variability was not formally assessed in this study; however, all molecular and histopathological analyses were performed under rigorously standardized conditions to ensure consistency and reliability. Pathology reports were meticulously reviewed to identify patients who had undergone molecular testing, with a focus on detecting critical genetic markers essential for glioma classification. Fluorescence in situ hybridization (FISH) analysis was utilized to determine the presence or absence of the 1p/19q codeletion, a key genetic alteration fundamental to differentiating oligodendrogliomas from other glioma subtypes. To ensure uniformity in FISH test results, all analyses were conducted using the same reagent kit (CytoCell Tissue Pretreatment Kit, Life Technologies Corporation, Carlsbad, CA, USA) and strictly adhered to the manufacturer's protocols. Additionally, all tests were carried out within a single institute, eliminating potential variability from inter-laboratory collaborations. By employing these standardized methodologies, the study ensured high accuracy and reliability in glioma differentiation, ultimately enhancing the precision of diagnostic and prognostic evaluations [9].

Immunohistochemistry (IHC) demonstrated the presence of mutations in *IDH* genes, specifically *IDH1* and *IDH2*, due to the relevance of these mutations in the context of glioma, as they often correlate with better outcomes and are indicative of treatment and tumor subtypes. Specifically, the co-occurrence of an *IDH1* mutation and a 1p/19q codeletion characterizes oligodendrogliomas, whereas the presence of an *IDH1* mutation without the 1p/19q codeletion is typical of astrocytoma [10].

FISH assays

The CytoCell Tissue Pretreatment Kit (catalog #LPS 100, Life Technologies Corporation, Cytocell Ltd., Carlsbad, CA, USA) was used following the manufacturer's guidelines.

Immunohistochemistry

The Universal HRP Immunostaining Kit (catalog #KP50, Diagnostic BioSystems, Pleasanton, CA, USA) identified *IDH1*/*IDH2* mutations, which are critical for glioma subtyping and prognostication. This information was obtained through a detailed review of the pathology report using the X-HIS Electronic Medical Record.

By integrating advanced imaging modalities with detailed molecular diagnostics, this study aims to refine the diagnostic accuracy for patients presenting with oligoastrocytoma and oligodendroglioma. This comprehensive approach ensures a deeper understanding of each patient's tumor type, facilitating more personalized treatment plans based on individual tumor characteristics. The synchronization of imaging data with genetic and histopathological insights is expected to drive forward the precision of medical interventions tailored to the unique profiles of gliomas.

Statistical analysis

In this study, the initial step involved performing the Kolmogorov-Smirnov test to evaluate the distribution of the collected data, ensuring the selection of appropriate statistical methods. Based on the test results, descriptive statistics were generated to summarize central tendencies and variability within the dataset.

Comparative analysis of numerical variables across patient groups was conducted using either the Mann-Whitney U test or Student's t-test, depending on the normality of the data distribution determined earlier. These tests provided insights into differences in key parameters, such as SUV max values, between subgroups.

Correlations between variables, including SUV max on MET PET scans, oligodendroglioma status, and the Ki-67 labeling index, were analyzed using Spearman's rank correlation coefficient. This analysis facilitated the identification of significant associations among critical clinical and imaging metrics.

For a more detailed exploration of the data, univariate and multivariate linear regression models were employed. These models evaluated the impact of independent variables, such as clinical and demographic factors, on the dependent variable (SUV max). Variables were selected based on their availability and potential relevance to patient outcomes. Statistical significance was defined as p < 0.05, and all significant findings were reported.

Additionally, a receiver operating characteristic (ROC) curve analysis was performed to determine the optimal SUV max cutoff for predicting the presence of the 1p/19q codeletion mutation. This analysis aimed to balance sensitivity and specificity, providing a robust diagnostic threshold for clinical application.

All statistical analyses were conducted using IBM SPSS Statistics for Windows, Version 25 (Released 2017; IBM Corp., Armonk, New York). This comprehensive statistical framework enabled a rigorous evaluation of correlations and relationships between clinical, imaging, and molecular parameters, enhancing the robustness and reliability of the study's findings.

## Results

This study included a cohort of 85 patients diagnosed with gliomas, with an emphasis on analyzing their demographic and clinical characteristics, as detailed in Table 1. Among the cohort, the majority of cases involved single lesions, predominantly localized to the right hemisphere (44.7%). The most commonly affected regions were the frontal and temporal lobes, each accounting for 22.4% of cases (19 patients per region). These were followed by the occipital, frontoparietal, and insular regions, each comprising 7.1% of cases (six patients per region). The corpus callosum was identified as the least frequently affected site, highlighting its rarity as a location for glioma occurrence.

**Table 1:** Demographic and clinical characteristics of the participants. KPS: Karnofsky performance scale; GTR: gross total resection; 1p/19q: codeletion 1p/19q; IDH1: isocitrate dehydrogenase type 1; IDH2: isocitrate dehydrogenase type 2; Ki-67: Ki-67 proliferation index; SUV max: maximal standardized uptake value; MGMT: unmethylated methylguanine methyltransferase.

Variable	Values			
	N			%
Median age (1–88 years)	46.8 years			
Female	44		51.80%	
Male	41		48.20%	
KPS	70%			
GTR	28		38.22%	
1p/19q	29		34.10%	
IDH1	47		47%	
IDH2	1		1%	
MGMT	16	18.8%		
Median Ki-67	8.49%			
Median overall survival (months)	78.8 months			
Median SUV max	3.70%			

Statistical analysis of SUV max differences

**Figure 1:**
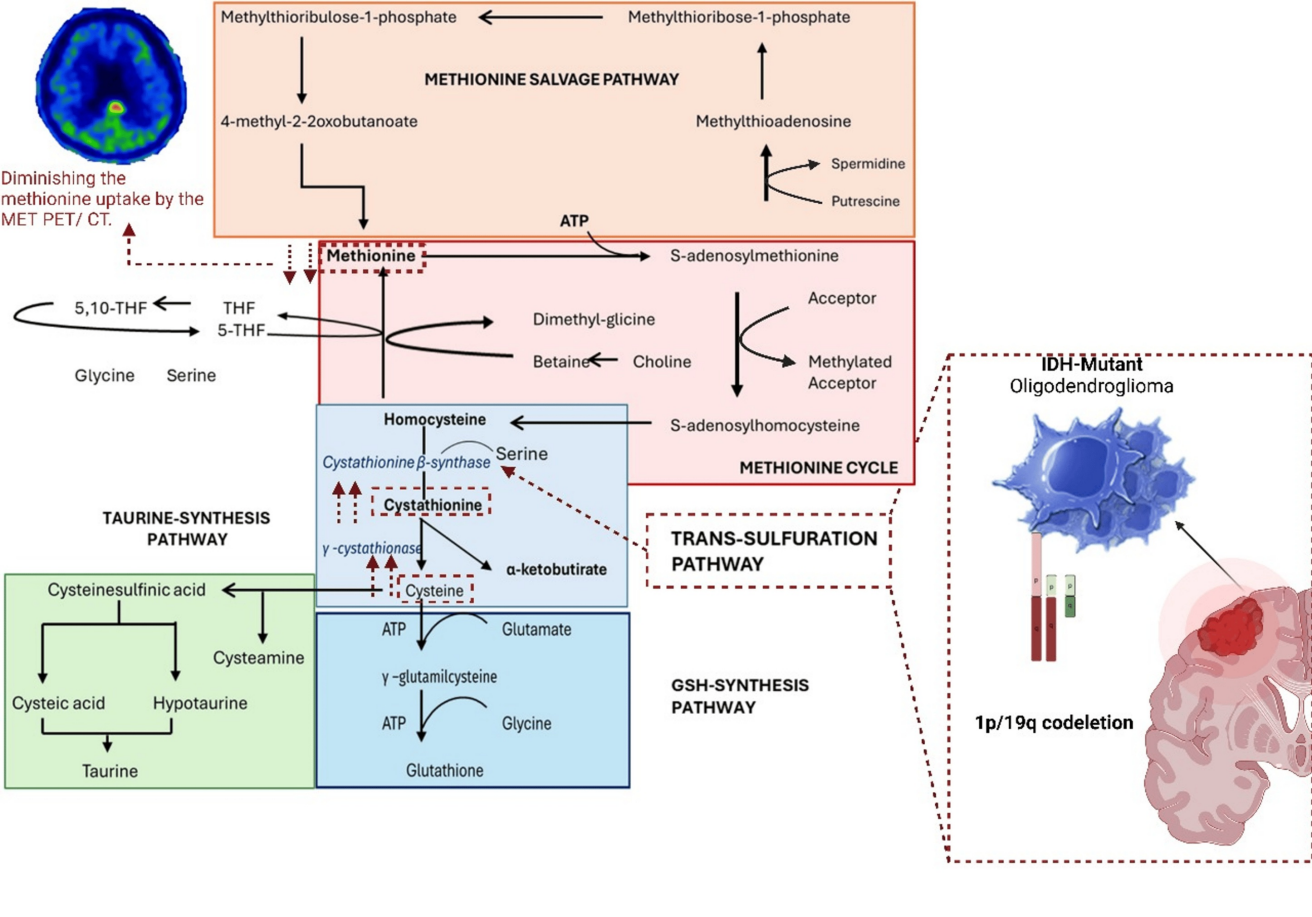
Metabolic alterations in oligodendrogliomas with mutated IDH and 1p/19q codeletion demonstrate impairment in the trans-sulfuration pathway, specifically through reduced activity of cystathionine beta-synthase. This disruption leads to diminished methionine uptake, as visualized by MET PET/CT imaging. THF: tetrahydrofolate; 5-THF: 5-methyltetrahydrofolate; 5,10-THF: 5,10-methylenetetrahydrofolate; ATP: adenosine triphosphate. Source: Adapted from the metabolic chart of biological sulfur compounds [11]. Image created with BioRender.com.

To evaluate differences in metabolic activity in gliomas, particularly focusing on the SUV max, a Mann-Whitney U test was employed. This analysis compared SUV max values between patients with oligodendrogliomas harboring the 1p/19q codeletion and those without it. The visual inspection of the SUV max distributions for both groups indicated comparable patterns; however, statistical testing identified significant differences. Additionally, the analysis revealed a notable disparity in median SUV max values based on gender (U = 189, z = -6.261, p < 0.0001), suggesting a gender-based variation in the metabolic activity of brain lesions. These findings provide insights into the metabolic heterogeneity of oligodendrogliomas and underscore the importance of considering demographic factors in the interpretation of PET imaging results (Figure 1).

Regression analysis on SUV max predictors

Further analysis employed multiple linear regression to identify predictors of SUV max. The absence of the 1p/19q codeletion and a high Ki-67 labeling index, indicative of elevated cellular proliferation, were identified as significant predictors of higher SUV max values (F-statistic = 47.40, p < 0.0001). Together, these factors accounted for 42.2% of the variability in SUV max within the patient cohort, underscoring their relevance in metabolic profiling.

Additionally, linear regression was applied to examine the interplay between the Ki-67 labeling index, 1p/19q codeletion status, and SUV max. The results demonstrated a clear linear relationship, illustrated by a scatterplot with a superimposed regression line. Statistical diagnostics confirmed the homoscedasticity and normality of residuals, affirming the reliability and robustness of the regression model. These findings highlight the importance of integrating molecular and proliferation indices into predictive imaging analyses for gliomas.

ROC curve analysis

The ROC curve analysis was utilized to determine the optimal SUV max cutoff for differentiating the presence or absence of the 1p/19q codeletion mutation, demonstrating robust diagnostic performance. The analysis revealed an area under the curve (AUC) of 0.840 (standard error: 0.055, p < 0.0001), underscoring the high diagnostic accuracy of SUV max in this context. An optimal cutoff value of 2.85 was identified, achieving a sensitivity of 91.1%, although with a false positive rate of 24.1%. This high sensitivity ensures reliable identification of true positives; however, the elevated false positive rate raises concerns about potential overdiagnosis, which could lead to unnecessary interventions or overtreatment in some cases.

To address this limitation, a cautious and integrative diagnostic approach is recommended. SUV max findings should be combined with molecular and histopathological assessments to enhance specificity and reduce the risk of diagnostic errors. This multimodal approach can provide a more comprehensive evaluation of glioma characteristics, improving diagnostic confidence and clinical decision-making. Despite its limitations, the chosen cutoff offers a practical balance between sensitivity and specificity, making it a valuable tool in the management of gliomas and aiding in the development of tailored treatment strategies.

**Figure 2:**
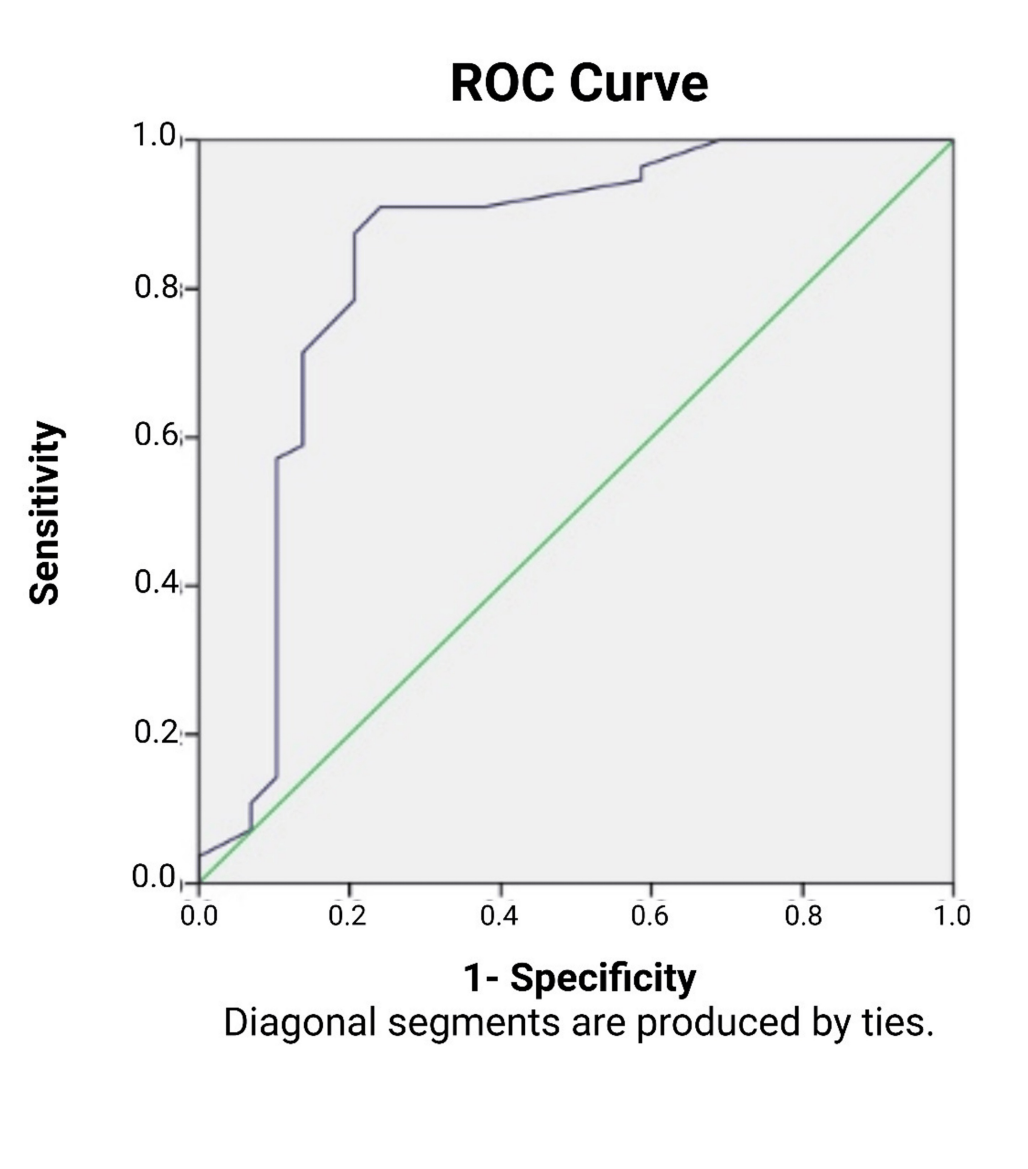
ROC curve and AUC for SUV max from MET PET and the detection of 1p/19q codeletion mutation. ROC: receiver operating characteristic; AUC: area under the curve; SUV max: maximum standardized uptake value; MET PET: positron emission tomography with 11C-labeled methionine.

These findings form a solid foundation for understanding the metabolic behavior of gliomas as captured by PET imaging metrics. By correlating SUV max values with key genetic and proliferative markers, this study highlights the potential of MET PET to guide clinical workflows, from initial diagnosis to treatment planning. Detailed analyses, as presented in Tables 2, 3 and Figure 2, provide deeper insights into these correlations and the statistical methodologies employed. This comprehensive approach not only advances the understanding of glioma pathophysiology but also underscores the pivotal role of PET imaging in enhancing diagnostic precision, optimizing therapeutic strategies, and ultimately improving patient outcomes.

**Table 2:** Univariable and multivariate linear regression analysis predicting SUV max levels. R^2^: coefficient of determination; B: unstandardized regression coefficient; SE: standard error of coefficient; Adj. R^2^: adjusted coefficient of determination; N/A: not applicable.

Variable	Univariable Linear Regression Analysis				Multivariate Linear Regression Analysis							
	R^2^	B	SE	P-value	Adj. R^2^	B	SE	P-value	Adj. R^2^	B	SE	P-value
Ki-67	0.123	0.109	0.032	0.001	0.422	0.084	0.026	<0.001	0.707	0.208	0.300	<0.001
1p/19q	0.437	-2.562	0.361	<0.001	0.409	-2.262	0.336	<0.001		-2.640	0.026	<0.001
IDH1	N/A	N/A	N/A	N/A	N/A	N/A	N/A	N/A		-2.640	0.319	0.099
IDH2	N/A	N/A	N/A	N/A	N/A	N/A	N/A	N/A		-2.820	1.133	0.015

**Table 3:** Coordinates of the ROC curve with sensitivity and false positive rate. ROC: receiver operating characteristic.

Positive if Greater Than or Equal to	Sensitivity	Specificity
0.20	1.000	1.000
1.25	1.000	0.966
1.45	1.000	0.793
1.65	1.000	0.759
1.75	1.000	0.690
1.85	0.964	0.586
1.95	0.946	0.586
2.05	0.929	0.483
2.15	0.911	0.379
2.30	0.911	0.310
2.60	0.911	0.276
2.85	0.911	0.241
2.95	0.875	0.207
3.05	0.839	0.207
3.15	0.786	0.207
3.30	0.714	0.138
3.44	0.625	0.138
3.49	0.589	0.138
3.55	0.571	0.103
3.65	0.554	0.103
3.75	0.518	0.103
3.85	0.446	0.103
4.05	0.429	0.103
4.25	0.375	0.103
4.35	0.357	0.103
4.45	0.339	0.103
4.65	0.304	0.103
4.90	0.250	0.103
5.05	0.232	0.103
5.45	0.214	0.103
6.15	0.143	0.103
6.60	0.107	0.069
6.95	0.089	0.069
7.70	0.071	0.069
8.70	0.054	0.034
9.25	0.036	0.000
10.30	0.000	0.000

## Discussion

The current study demonstrates a significant correlation between the absence of the 1p/19q codeletion, elevated Ki-67 levels, and increased mean SUV max on MET PET scans, emphasizing the diagnostic utility of MET PET in glioma evaluation. This advanced imaging modality is instrumental not only in distinguishing tumors based on 1p/19q codeletion status but also in assessing tumor metabolic activity, an essential component for personalized therapeutic planning.

An optimal SUV max cutoff of 2.85 was identified, achieving a sensitivity of 91.1% but with a false positive rate of 24.1%, necessitating cautious interpretation to avoid overdiagnosis and overtreatment. To mitigate these risks, integrating MET PET findings with complementary diagnostic modalities, such as advanced MRI, molecular testing (e.g., *IDH* mutations and 1p/19q codeletion analysis), and histopathological evaluations, is essential. These additional diagnostic layers, combined with standardized imaging protocols and clinical context, enhance specificity, reduce unnecessary interventions, and improve the overall accuracy of treatment decisions, ensuring a comprehensive understanding of the tumor's biological and metabolic characteristics.

Chromosomal deletions of 1p and 19q are observed in 60-90% of oligodendroglial tumors and 30-50% of oligoastrocytomas [12]. This mutation is associated with improved survival rates and increased chemosensitivity. Additionally, the 1p/19q deletion is uniquely linked to *p53* overexpression or the presence of MGMT [12].

Amino acid uptake in tumor cells occurs primarily via a sodium-dependent transport system in the cell membrane, which is overexpressed in tumor cells and correlates with their growth rates. The activity of this transport system is influenced by several factors, including pH, hormones, growth factors, amino acid availability, and the rate of cellular proliferation [13]. Elevated methionine uptake in oligodendrogliomas is largely attributed to increased cell density, which drives more intensive transport system activity within tumor tissues. Additionally, increased microvessel density in oligodendrogliomas may enhance methionine uptake through angiogenesis, promoting the carrier-mediated transport of amino acids in large quantities.

Wang et al. identified a pronounced differential expression of 45 metabolism-associated genes across various glioma histological types. Astrocytomas, for instance, exhibited elevated levels of glycolysis-related proteins such as hexokinase 2, lactate dehydrogenase A (LDHA), and glucose-6-phosphate dehydrogenase compared to oligodendrogliomas [14]. Furthermore, the impact of heterozygous deletions on chromosomes 1p and 19q has been explored in limited studies, revealing potential mutations that may influence cell metabolism and the tumor microenvironment. These mutations include alterations in phosphoglycerate dehydrogenase (PHGDH), cystathionine gamma-lyase (CTH), and sodium-hydrogen exchanger 1 (NHE) on 1p, as well as capicua transcriptional repressor (CIC) on 19q [14].

The present study builds on the findings of Yao et al., who demonstrated that magnetic transfer ratio asymmetry on MRI and F-FDOPA PET could effectively differentiate 1p/19q codeletion status in gliomas, achieving an AUC of 0.85 [12]. Their work linked lower extracellular acidity to better prognoses, mediated through mechanisms such as NHE-1 silencing and CIC mutations. While Yao et al. primarily focused on extracellular acidity, our study emphasizes the metabolic profiles of gliomas, utilizing MET PET to establish an SUV max cutoff of 2.85 with high sensitivity (91.1%) for identifying tumors lacking the codeletion. By correlating these metabolic variations with Ki-67 levels, our findings complement Yao et al.'s biochemical insights, underscoring the importance of integrating metabolic, genetic, and molecular markers to improve glioma diagnosis and therapeutic planning.

Branzoli et al. provided further insights by identifying cystathionine accumulation in vivo through magnetic resonance spectroscopy in 31 subjects with glioma. Their findings showed statistically significant elevations of cystathionine levels in *IDH*-mutated, 1p/19q-deleted gliomas, while healthy tissues exhibited no detectable levels. This phenomenon was attributed to the lower expression of PHGDH and CTH, enzymes involved in serine and cystathionine metabolism, in gliomas with chromosomal alterations compared to those without them [15].

Additionally, cystathionine and cysteine concentrations were markedly higher in *IDH*-mutated, 1p/19q-deleted samples compared to *IDH*-mutated non-deleted samples. In the latter group, cystathionine correlated with methionine levels, while in wild-type samples, cystathionine was linked to metabolites associated with the Krebs cycle and glutamine metabolism. This suggests that methionine may accumulate in higher concentrations in gliomas lacking the aforementioned alterations [14]. These observations were corroborated by two independent studies, which demonstrated significantly lower mean mRNA levels for PHGDH in *IDH*-mutated, 1p/19q-deleted gliomas compared to *IDH*-mutated, non-deleted gliomas [16,17].

These findings provide a plausible explanation for the elevated MET uptake observed in gliomas without the 1p/19q codeletion, reflected in higher SUV max values compared to gliomas with the codeletion. In our study, the* IDH-1* mutation exhibited a significant correlation with higher SUV max values, as demonstrated in the multivariate regression model (Table 2).

In 2015, Iwadate et al. investigated 144 patients with histologically confirmed gliomas who underwent MET PET imaging between 2002 and 2014. This imaging modality was subsequently used for navigated resections and detailed tumor grading. Their findings highlighted that the mean SUV tumor-to-normal (T/N) ratio obtained via MET PET was significantly higher in oligodendroglial tumors without the 1p/19q deletion compared to those with the deletion, further validating the utility of this imaging approach in glioma characterization [18].

In 2011, Shinozaki et al. investigated the clinical utility of MET PET in gliomas, specifically its role in the preoperative diagnosis of histological type and grade. Their study of 70 patients with histologically confirmed intracerebral gliomas demonstrated that the T/N ratio significantly increased with advancing tumor grade in astrocytic tumors. Among grade 2 gliomas, oligodendroglial tumors exhibited a notably higher mean T/N ratio compared to diffuse astrocytomas. Additionally, they observed a strong correlation between the Ki-67 labeling index and the T/N ratio in astrocytic tumors, although this association was not evident in oligodendrogliomas [13].

In 2020, Ogawa et al. compared the diagnostic performance of PET/CT using MET and 18F-fluorothymidine (FLT) in newly diagnosed gliomas. Their findings focused on the diagnostic accuracy for detecting *IDH1* mutations [9]. This contrasts with our study, which identified a statistically significant correlation between Ki-67 and SUV max values associated with 1p/19q codeletion status using MET PET [12].

Kim et al. reported paradoxically higher MET uptake in grade 2 and 3 gliomas with *IDH1* mutations and 1p/19q codeletions compared to *IDH1*-wildtype astrocytomas [19]. In contrast, our analysis revealed reduced uptake when the 1p/19q codeletion was present. Similarly, Zhao et al. observed that gliomas with higher histologic grades, *IDH1*-wildtype status, and the absence of 1p/19q codeletion displayed increased MET uptake [20]. These findings suggest that the 1p/19q codeletion may influence downstream pathways, altering gene expression and microvascular architecture in oligodendrogliomas [19,20].

Beyond MET PET, other PET scan modalities have also been explored. In 1996, Goldman et al. analyzed 161 biopsy samples from 20 PET-guided procedures in glioma patients using 18F-2-fluoro-2-deoxy-D-glucose (FDG) [22]. Their study revealed differences in glucose metabolic rates between anaplastic and non-anaplastic samples and among metabolic grades normalized to cortical or white matter values.

Strategies for imaging biological tissue characteristics, including the uptake of metabolic substrates such as glucose, amino acids, and nucleotides, have proven valuable in noninvasive studies of physiological functions and pathological lesions [12,21]. This methodology has also been useful in detecting low-grade gliomas when conventional imaging modalities provide inconclusive results, as it effectively distinguishes between low-grade and high-grade gliomas.

The 2021 WHO classification of brain tumors introduced a refined framework that integrates morphological and molecular characteristics, enhancing the precision of tumor categorization, including for oligodendrogliomas. This classification emphasizes comprehensive molecular assessments, particularly focusing on *IDH* mutations and the codeletion of chromosome arms 1p and 19q [22,23]. These molecular criteria are pivotal for accurate tumor classification and play a critical role in tailoring treatment strategies and prognostic evaluations.

The application of MET PET has transformed the clinical management of gliomas by offering critical insights into their metabolic behavior. This imaging modality is instrumental not only in the initial diagnosis but also in the ongoing monitoring of gliomas, enabling dynamic adjustments to treatment plans based on metabolic activity. MET PET's ability to detect subtle metabolic changes provides a distinct advantage in distinguishing tumor types, evaluating therapeutic efficacy, and identifying early signs of recurrence, ultimately enhancing patient outcomes.

In resource-limited settings, such as our institution, challenges, including budget constraints and the lasting effects of the COVID-19 pandemic, have delayed critical surgical interventions. These delays are particularly detrimental in managing oligodendrogliomas, where timely treatment is essential for optimal outcomes. To address these barriers, strategies such as implementing awake craniotomy to reduce resource dependency, utilizing telemedicine for efficient triage and decision-making, and standardizing imaging and histopathological protocols are recommended. Task-sharing initiatives to train healthcare providers in neurosurgical skills further enhance care delivery. Supported by findings from recent studies on awake craniotomy in low-resource environments [24], these measures offer practical and cost-effective solutions for improving outcomes in glioma care.

Expanding the implementation of MET PET imaging represents another effective strategy for addressing resource limitations while improving diagnostic precision. This advanced imaging modality provides high diagnostic accuracy and specificity, facilitating the prioritization of patients for surgical and therapeutic interventions. By optimizing treatment schedules, MET PET enhances the precision of glioma management and improves the efficacy of radiotherapy and chemotherapy. Particularly in resource-constrained settings, MET PET mitigates delays in care and ensures timely, effective treatment, contributing to better clinical outcomes.

The integration of quantitative MET PET imaging with molecular and histopathological analyses establishes a robust framework for advancing clinical evaluations. This multidisciplinary approach improves diagnostic accuracy, supports personalized therapeutic strategies, and enhances the overall management of oligodendrogliomas. By leveraging these complementary methodologies, clinicians can refine prognostic assessments, optimize therapeutic interventions, and achieve improved outcomes for glioma patients.

While this study highlights key strengths, including the integration of metabolic imaging and molecular diagnostics, several limitations require careful consideration. The retrospective design and limited sample size constrain the generalizability of findings, particularly given the inclusion of both pediatric and adult patients, whose metabolic and genetic profiles may vary significantly. Furthermore, reliance on data from a single institutional cohort without external validation restricts the broader clinical applicability of these results. To address these limitations, future research should prioritize larger, multicenter cohorts, incorporating age-stratified analyses and standardized imaging protocols to ensure a more comprehensive evaluation. Prospective, multicenter trials that leverage advancements in imaging technology and molecular profiling will be crucial for enhancing reproducibility and clinical relevance. Such trials would facilitate diverse patient representation, standardize methodologies across institutions, and validate the generalizability of findings across varied clinical environments, ultimately strengthening the applicability of these results to a broader patient population.

## Conclusions

This study underscores the potential diagnostic and clinical utility of MET PET in glioma management, particularly its ability to differentiate metabolic profiles associated with the 1p/19q codeletion. While the findings support its role in assessing glioma metabolic activity, the study's retrospective design, small sample size, and single-center cohort limit the generalizability of the conclusions. To build on these preliminary findings, prospective, multicenter studies with larger and more diverse cohorts are crucial. Such investigations should aim to validate the observed relationships between tracer uptake, WHO malignancy grades, and key genetic alterations, including *IDH1* and *IDH2* mutations, to enhance the prognostic and diagnostic capabilities of MET PET.

Beyond diagnostics, MET PET demonstrates the potential for longitudinal glioma management by detecting early tumor recurrence and informing therapeutic adjustments. However, its broader clinical utility must be confirmed through future studies that evaluate its application across diverse settings. While its sensitivity to metabolic changes makes it a promising tool, particularly in resource-constrained environments, its integration into standard clinical protocols requires further validation. By facilitating precise diagnoses, informed surgical planning, and ongoing monitoring, MET PET could contribute significantly to personalized glioma care, pending robust prospective evidence to support its widespread adoption.
